# Leaf Anatomy and Micromorphology Characteristics of Ketum [*Mitragyna speciosa* (Korth.) Havil.] (Rubiaceae)

**DOI:** 10.21315/tlsr2021.32.1.7

**Published:** 2021-03-31

**Authors:** Mohd Norfaizal Ghazalli, Muhammad Shafie Md Sah, Mazidah Mat, Khadijah Awang, Mohd Azwan Jaafar, Razali Mirad, Ahmad Zaki Zaini, Anuar Rasyidi Mohd Nordin, Nurshahidah Mohd Rusli, Siti Sofiah Mohamad, Ahmad Syahman Mohd Dalee

**Affiliations:** 1 Resource Utilisation and Agrobiodiversity Conservation Programme (BE2), Agrobiodiversity and Environment Research Centre, MARDI Headquarters, 43400 Serdang, Selangor, Malaysia; 2 Pest and Diseases Programme (IC3), Industrial Crops Research Centre, MARDI Headquarters, 43400 Serdang, Selangor, Malaysia; 3 Breeding Programme (IC1), Industrial Crops Research Centre, MARDI Headquarters, 43400 Serdang, Selangor, Malaysia; 4 Agri-Omics and Bioinformatics Programme (BN1), Biotechnology and Nanotechnology Research Centre, MARDI Headquarters, 43400 Serdang, Selangor, Malaysia; 5 Electron Microscopy Unit, Faculty of Sciences and Technology, Universiti Kebangsaan Malaysia, 43600 Bangi, Selangor, Malaysia

**Keywords:** *Mitragyna speciosa*, Rubiaceae, Ketum, Anatomy, Micromorphology, *Mitragyna speciosa*, Rubiaceae, Ketum, Anatomi, Mikromorfologi

## Abstract

*Mitragyna speciosa* (Korth.) Havil. or locally known as ketum/daun sebiak/biak-biak belongs to Rubiaceae family and generally occurs in secondary forest or disturbed areas in tropical and subtropical region. This research enumerated the characterisation of *Mitragyna speciosa* leaf anatomy and micromorphology features which is still not well documented. This medium to large sized tree species characterised with opposite arrangement, ovate-acuminate leaf and with 12–17 pairs of veins. Transverse sections of petioles showed that this species has petiole outlines with slightly convex at the middle of the adaxial part and ‘U’-shaped on abaxial side. Results also showed that this species has paracytic and hypostomatic stomata, combination of non-glandular (majority) and glandular trichomes (minority), with observation on the secretory cells present in petiole and midrib parenchyma cells. Cuticle on the abaxial and adaxial epidermal surfaces showed the presence granule and wax films with periclinal and anticlinal walls can be differentiated clearly. The results obtained in this study can be used to providing additional systematics information of *Mitragyna speciosa* with the documentation of the leaf anatomy and micromorphology characters.

## Abstract (ms)

*Mitragyna speciosa* (Korth.) Havil. atau dikenali dengan nama tempatan sebagai ketum/daun sebiak/biak-biak dikelaskan di dalam famili Rubiaceae dan kebiasaannya boleh dijumpai di kawasan hutan sekunder atau tanah terbiar di wilayah tropika atau subtropika. Kajian ini menghuraikan ciri-ciri anatomi daun dan mikromorfologi *Mitragyna speciosa* yang masih lagi belum didokumenkan secara terperinci. Spesies ini merupakan pokok bersaiz sederhana ke besar dengan susunan daun bertentangan, daun berbentuk ovat-akuminat dengan 12–17 pasang urat daun. Keratan rentas petiol menunjukkan ciri bentuk luaran sedikit cembung pada bahagian tengah adaksial dan berbentuk ‘U’ pada bahagian abaksial. Kajian ini juga menunjukkan spesies ini mempunyai stomata jenis parasitik, hipostomatik, kombinasi trikom bukan kelenjar (majoriti) dan trikom berkelenjar (minoriti), dengan pemerhatian sel rembes pada bahagian sel parenkima petiol dan tulang daun. Kutikel pada epidermis abaksial dan adaksial menunjukkan kehadiran butir granul dan filem lilin dengan dinding periklinal dan antiklinal yang boleh dibezakan dengan jelas. Pemerhatian melalui kajian ini memberikan maklumat tambahan sistematik *Mitragyna speciosa* melalui dokumentasi ciri anatomi dan mikromorfologi daunnya.

**Highlights**

- First study to enumerates the anatomy and micromorphology characteristics of *Mitragyna speciosa* (ketum/sebiak) collected from Peninsular Malaysia.
- This finding highlighted the observation of paracytic and hypostomatic stomata, non-glandular and glandular trichomes, with observation on the secretory cells present in petiole and midrib parenchyma cells which complement the basic study by Metcalfe and Chalk (1950).
- Additional information on systematic morphology feature of *M. speciosa*.

## INTRODUCTION

Rubiaceae is considered as major plant families in tropical region, recorded over 13,000 species that consists of vines, small herbs, shrubs and large-sized trees (Govaerts *et al.* 2013; Timothy & Gemma 2014). Also known as coffee or madder family, this family includes minor genus of *Mitragyna* that can be found in swampy and valleys of tropical and subtropical Asia, i.e., Thailand, Laos, Cambodia and Malaysia (Burkill 1935; Havala & Hinou 1988). This genus primarily can be found thriving very well in damp areas with rich humus in Old World Tropics region (Macko *et al.* 1972).

**Figure 1:**
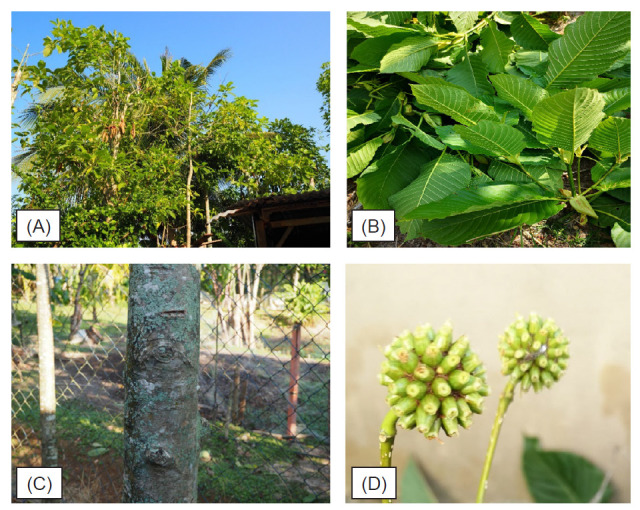
*M. speciosa* morphology. (A) Habit; (B) Leaves (ketum urat hijau); (C) Trunk – straight and greyish in colour; (D) Inflorescences.

According to Burkill (1935), there are three species of *Mitragyna*, namely, *M. rotundifolia, M. parvifolia* and *M. speciosa.* Out of these, *M. parvifolia* and *M. rotundifolia* are well known for its medicinal properties and *M. speciosa* is the most potent species in terms of its alkaloid contents (Sosef *et al.* 1998). In Peninsular Malaysia, *M. speciosa* can be found thriving in the secondary forests or nearby the abandoned paddy field and forest ridges in Perak, Selangor, Pahang and Kelantan as well as in the northern region (Ng 1989). In spite of the medicinal properties and useful characters, systematic documentation of *Mitragyna* has not been given much emphasis. In addition, the distribution and genetic diversity of those *Mitragyna* species in Malaysia that is expected to have significant large genetic diversity is also not sufficiently investigated. *M. speciosa* or ketum (Fig. 1) is synonym with the presence of chemical constituents monoterpenoid indole – alkaloids that traditionally used in traditional folk medicine for diarrhea, fever, asthma, antihypertensive, pain reliever and also for morphine substitute in medical-field purposes (Zacharis *et al.* 1964; Matsumoto *et al.* 1996; Reanmongkol *et al.* 2007). Mitragynine (MGN) is the active compund that derived from the species *M. speciosa*, can be utilised into potential pharmaceutical application (Jagabalan *et al.* 2019; Suhaimi *et al.* 2016).

Considering the importance of this species in potential medicinal purpose, it is important to document and enumerating its anatomy and micromorphology information while understand it forms for species identification and physiology functioning analysis (Noraini & Cutler, 2009; Mohd Norfaizal & Latiff 2014). This present study investigated the systematic anatomy and its documentation of *M. speciosa* that were collected from different localities in Peninsular Malaysia.

## MATERIALS AND METHODS

### Leaves Anatomy Observation

Fresh specimens used for this study were obtained from various localities from Peninsular Malaysia during the intensive fieldwork conducted between February to April 2019 (Table 1). Fresh leaves were fixed in combination of AA solutions (1 acetic acid:3 alcohol) with 3–5 matured leaves were sectioned from each voucher specimen collected from the field. Materials were sectioned in a range of thickness (15 μm–45 μm) using sliding microtome. Transverse sections were taken from the middle part of matured leaves. The sections then were cleared using bleaching agent, washed and stained in Safranin and Alcian Green for 5 min each. They were then dehydrated through an ascending alcohol series prior to differentiated in 70% alcohol with a drop of hydrochloric acid. The slides were kept in the oven for nine days at about 60°C. Photographs of the sections were taken using Olympus SZH40 microscope (Tokyo, Japan) and anatomical images were processed using Image Analysis and ADOBE Photoshop Software. Leaves samples fixation followed the methods of Johansen (1940) with a few technical preparation modifications.

### Leaf Micromorphology Observation

Sections of 3 mm^2^–5 mm^2^ of the matured leaf were prepared and soaked in a mixed solution of 8% glutaraldehyde and Sorensen’s phosphate buffer with a ratio 1:1 for 1 h. Using a combination of Sorensen’s phosphate buffer solution and distilled water in a ratio 1:1, leaves samples were washed for 5 min before soaked in a mixed solution of 4% Osmium and distilled water (1:1) for another 14 h at low temperature (4°C). Later, the specimens soaked in ascending methanol series (10% to 100%) for every 15 min in each concentration. Specimens were then soaked in a mixture of 100% ethanol and 100% acetone with ratios 3:1, 1:1 and 1:3 for 20 min, respectively. Specimens were then soaked in 100% acetone solution for 20 min, with continuous repeating for four times. Next, CO_2_ liquid added in order to flush the solvent from the tissue using Critical Point Drying (CPD) methodology. The coated specimens with gold by using BIO-Rod SEM Coating System were examined under JEOL JSM-7001F Field Scanning Electron Microscope (FESEM, Tokyo, Japan) for each labelled stub and mounted by using Conducting Carbon Cement (LEIT-C, Essex, UK) to ensure conductive continuity.

## RESULTS

**Figure 2:**
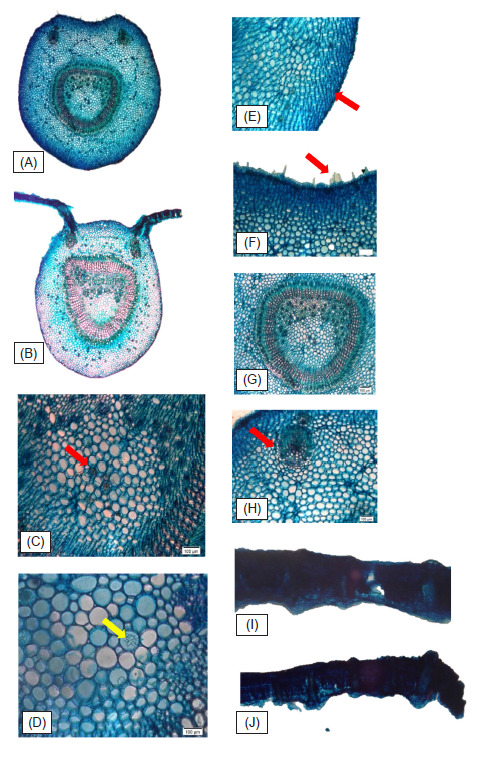
Transverse sections of *M. speciosa*. (A) Petiole TS; (B) Midrib TS; (C, D) Druses (Arrow); (E) Collenchyma cells (arrow); (F) Simple, unicellular trichomes observed on the adaxial side of the petiole TS; (G) Vascular bundle; (H) Additional vascular bundle; (I) Lamina TS; (J) Margin TS. (Scale: A, B = 500 μm, C, D, E, F = 100 μm, G, H = 200 μm, I, J = 200 μm.)

Leaf anatomy and micromorphology features for *M. speciosa* was analysed and recorded both under Light Microscope (LM) and Scanning Electron Microscope (SEM) and their observation notes showed in Figs. 1 and 2, while their characters documented in Tables 2 and 3. Enumeration on the anatomy and micromorphology characters includes thin epidermis cuticular layer, single to double layers of adaxial and abaxial epidermis part. The presence of capitate glandular trichomes on the abaxial surface while simple and unicellular trichome were observed on the adaxial surface. Micromorphological features of both adaxial and abaxial leaf surfaces under SEM for the species are hypo-stomatic, and stomata are dispersed densely all over the leaf abaxial surface with broad-elliptic in shape and arisen on the abaxial epidermis surface.

## DISCUSSION

Results of this analysis showed important anatomical characteristics of *M. speciosa* documented. Druses are present in enlarged cells of the palisade layer and also in parenchyma cells of the petiole and midrib as well as in the phloem cells, supported Metcalfe and Chalk (1985) that noted the presence of raphide, druse, styloid, prismatic, and sand-like crystal idioblasts in species of the Rubiaceae (Figs. 2C and 2D). Presence and distribution in combination with type of prismatic, druse or styloid crystal can be used as a taxonomic character. Small crystals observation in higher plants is common and is related to physical protection, removal of oxalate from the metabolic system and helps in regulation of light during photosynthesis in plants that grow in the shade (Franceschi & Nakata 2005).

**Figure 3:**
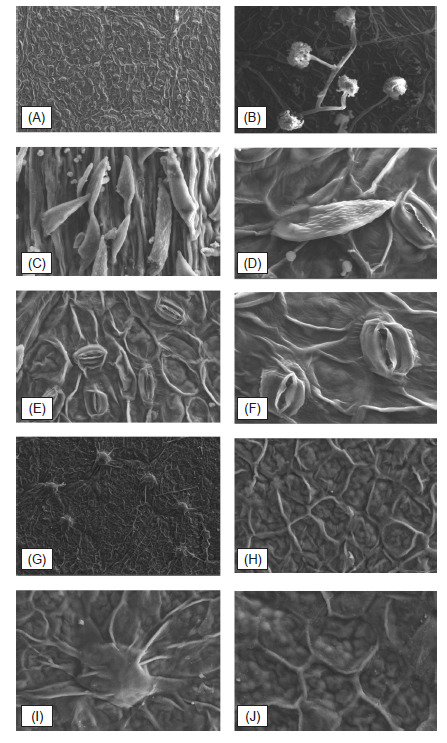
SEMs of adaxial and abaxial surfaces of leaf showing the surface sculpturing and the types of stomata of *M. speciosa*. (A) Abaxial surface; (B) Capitate glandular trichome; (C, D) Simple unicellular trichome with echinate ornamentation; (E, F) Arisen stomata; (G) Adaxial surface; (H) Adaxial surface ornamentation; (I) Wart; (J) Adaxial surface ornamentation (close up). (Scale: A, G, H : 10 μm; B, C, D, E, F, I, J: 2 μm.)

Four to six layers of collenchyma cells was observed in the studied *M. speciosa* midrib and petioles transverse section, supporting previous study by Metcalfe and Chalk (1950) involving several important genera in Rubiaceae family (Fig. 2E). Paracytic stomata with hypostomatic was observe generally in this species, in which these characters were discussed earlier by (Metcalfe & Chalk 1950, 1979) from his basic survey on the Rubiaceae and described that hypostomatic stomata is one of general characteristics of this family members (Figs. 3E and 3F). Observation on the underside of the lamina, midrib and petiole transverse sections showed the presence of simple, unicellular trichome in combination with capitate glandular trichome (Figs. 2A, 2B and 2F). The presence or absence of trichomes in a species can be a diagnostic character used for identification. Rubiaceae family appendages characters can be relatively simple structures (Robbrecht 1988). According to Metcalfe and Chalk (1950), they may be unicellular, uniseriate, in tufts, or rarely stellate. According to Solereder (1908), simple, unicellular hairs are more frequent in Rubiaceae family in comparison with glandular hairs, specifically on the adaxial parts of the lamina and leaves parts.

The vascular bundle in the midrib and petiole of *Mitragyna speciosa* characterised with closed system and consists of one continous vascular tissue in “0” shape (Figs. 2A and 2G). Transverse sections also showed that the main vascular bundles was enumerated with two additional or accessory vascular bundles at the left and right side of the adaxial part (Figs. 2A, 2B and 2H). The organisation of the vascular system of the midrib is the same as the petiole (Fig. 2B). The support tissue is represented by angular collenchyma (Fig. 2A), adjacent to the epidermis in both sides and to the sheath of fibers that surrounds the vascular system. Besides the primary bundle, the presence of 1–3 accessory bundles was noted in majority of Rubiaceae species with conductive elements of the xylem were in a radial series separated by cells of parenchyma (Metcalfe & Chalk 1950).

Epidermal surface analysis has been useful in defining and helps in characterise several plant families, genera and species, such as in Pinaceae (Whang *et al.* 2004) and Myrtaceae (Fontenelle *et al.* 1994). Under the examination using Scanning Microscopy Electron (SEM), the surface sculpturing is slight striae and undulate (Figs. 3A and 3G). In this study of *Mitragyna speciosa*, physically the slight striae-wavy part can be observed on the abaxial and adaxial surfaces (Fig. 3A). Barthlott (1981) stated that morphological aspects of the leaf surface, such as the presence or absence of cuticular ornamentations, can be used to separate taxa at the genus and species level. The epidermal cells of the leaf surfaces in the species examined is irregular in shape with epicuticular wax occurs on the leaf surfaces in the form of granule and wax film (Figs. 3A, 3G and 3H). Simple, unicellular trichomes (Figs. 3C and 3D) were densely distributed on the leaf base surface, with capitate glandular trichome can be observed mainly on the part nearby vein segmentation of the lamina (Fig. 3B). Indeed, distibution pattern of the trichomes in plants are important as proved by Xiang *et al.* (2010) that reported the morphology and distribution of trichomes have valuable taxonomic significance at species level in *Chelonopsis* (Lamiaceae). However, further studies are recommended to determine whether these features are influenced by environmental factors or may be under genetic control.

We do conclude that the anatomy and micromorphology of the leaves of the *M. speciosa* studied could be used in adding more systematic data on this species which is still lack in this type of information. The characters that contributed to the formation of the groups were a petiole with slightly convex at the middle of the adaxial part; U-shaped on the abaxial part, the presence of granular and wax film layers on the abaxial and adaxial surfaces of the lamina, presence of warts on abaxial surface only (SEM) and observation of simple, unicellular trichome in combination with capitate glandular trichome (unicellular) on abaxial and adaxial epidermis surfaces.

## CONCLUSION

As a conclusion, enumeration on the anatomy and micromorphology characteristics of *M. speciosa* helps in adding important significance systematic information of this species with some of the characteristics are agreeable with previous studies conducted among other Rubiaceae members. However, further studies involving other *Mitragyna* taxa in Malaysia are highly recommended to complement the analysis and systematic compilations of this genus.

## Figures and Tables

**Table 1:** Selected collection sites of *M. speciosa* in Peninsular Malaysia.

Taxon	Localities
*M. speciosa (*Korth.) Havil.	Kg. Permatang Bungor, Kota Sarang Semut, Kedah. Mohd Norfaizal G. & Salmaniza S. MDI12452, 19.2.2019. (MDI).
	Kg. Simpang Geti, Mata Ayer, Perlis. Mohd Norfaizal G. & Salmaniza S. MDI12453, 21.2.2019. (MDI).
	Kg. Sawah Ring, Muar, Johor. Muhammad Shafie Md. Sah, Anuar Rasyidi M N & Ahmad Syahman M D. MDI12454, 25.2.2019. (MDI).
	Kg. Lonek, Bahau, Negeri Sembilan. Muhammad Shafie Md. Sah, Anuar Rasyidi M N & Ahmad Syahman M D. MDI12455, 1.3.2019. (MDI).
	Kg. Merbau Menyusut, Setiu, Terengganu. Mohd Norfaizal G, Salmaniza S & Nurshahidah M R. MDI12456. 6.3.2019. (MDI).
	Kg. Ayer Chanal, Tanah Merah, Kelantan. Mohd Norfaizal G, Salmaniza S & Nurshahidah M R. MDI12457. 5.3.2019. (MDI).
	Pekan, Pahang. Mohd Norfaizal G, Salmaniza S & Nurshahidah M R. MDI12458. 7.3.2019. (MDI).
	Kg. Bukit Kuching, Jeram, Selangor. Anuar Rasyidi M N, Ahmad Syahman M D & Siti Sofiah M. MDI12459. 11.3.2019. (MDI).
	Kg. Luat, Lenggong, Perak. Anuar Rasyidi M N, Ahmad Syahman M D & Siti Sofiah M. MDI12460. 13.3.2019. (MDI).

**Table 2:** Leaf anatomical characteristics of *M. speciosa.*

Characteristics	Observation
Leaf lamina	Cuticular layer: relatively thin. Adaxial epidermis: single layer with height:width ratio – 1:3. Abaxial epidermis: single layer with height:width ratio – 1:2. Chlorenchyma cells: mesophyll palisade: single layer filling ¾ part of the height of leaf lamina. Spongy mesophyll: 2–3 layers of spongy mesophyll. Vascular bundles: simple vascular bundles. Parenchyma cells: single layer encirling each vascular bundle. Trichome: not observed.
Leaf margin	Outline: slight tapered, 60° recurved downwards to the abaxial side.
Petiole	Adaxial side: slightly convex at the middle of the adaxial part; abaxial side: U-shaped. Epidermal cells: ratio height:width (2:1). Main vascular tissue system – closed system, consists of one continous vascular tissue in “O” shape. Palisade cells: present underneath epidermis cells at adaxial side. Vascular tissue: main vascular tissue (closed system with continuous ring of vascular bundle in “O” shape with two additional vascular bundles are situated at the above left and right side of the main vascular bundle. Schlerenchyma cells: cluster of schlerenchyma cells are densely scattered around the vascular bundle. Parenchyma cells: parenchyma cells at the medullary area of the petiole are larger in size than other parenchyma cells. Collenchyma cells: 2–4 layers of collenchyma cells present underneath epidermis cells surroundings the petiole. Cells inclusion: secretory cells present in medullary cortex area. Trichome: simple, unicellular trichome present mainly at the adaxial part of the petiole.
Midrib	Outline: adaxial: slightly convex at the middle; abaxial side: broad U-shaped. Vascular tissue: main vascular tissue (closed system with continuous ring of vascular bundle in ‘O’ shape with two additional vascular bundles are situated at the above left and right side of the main vascular bundle. Schlerenchyma cells: cluster os of schlerenchyma cells are densely scattered around the vascular bundle. Collenchyma cells: 2–4 layers of collenchyma cells present underneath epidermis abaxial and adaxial. Cells inclusion: druses and secretory cells present in pith parenchyma and parenchyma cortex. Trichome: simple unicellular trichome present mainly at the adaxial side.
Trichome	Simple, unicellular (short, pointed end) Capitate glandular trichome

**Table 3:** Leaf epidermis micromorphological characteristics of *M. speciosa.*

Characteristics	Observation
Cuticular striation	Adaxial and abaxial epidermis: cuticular striation obscuring, anticlinal and periclinal walls can be differentiated directly. Fine striae present on the abaxial and adaxial epidermis surfaces.
Epicuticular waxes	Granules Film layers of wax present on both adaxial and abaxial epidermis
Stomata feature	Hypostomatic, superficial, densely scattered on the abaxial epidermis surface. Broad elliptical shape. Stomata size: L (17.58 μm–18.90 μm) × W (12.01 μm–13.58 μm).
Trichomes	Mainly on the adaxial surface; sparsely distributed on the abaxial surface. Type: Simple unicellular trichome (long, pointed tip with echinate ornamentation) Capitate glandular trichome (unicellular terminal)
